# Thermosensitive alginate–gelatin–nitrogen-doped carbon dots scaffolds as potential injectable hydrogels for cartilage tissue engineering applications

**DOI:** 10.1039/d1ra01496j

**Published:** 2021-05-21

**Authors:** Mojgan Ghanbari, Masoud Salavati-Niasari, Fatemeh Mohandes

**Affiliations:** Institute of Nano Science and Nano Technology, University of Kashan P. O. Box. 87317-51167 Kashan I. R. Iran salavati@kashanu.ac.ir +98 31 55913201 +98 31 5591 2383

## Abstract

Hybrid injectable and biodegradable hydrogels based on oxidized alginate/gelatin and containing nitrogen-doped carbon dots (NCDs) as a reinforcement have been fabricated and crosslinked by 1-ethyl-3-(3-dimethylaminopropyl) carbodiimide (EDC)/*N*-hydroxysuccinimide (NHS) as the chemical crosslinking agents in the hydrogel system. The idea of composite hydrogels relies on the assumption that they supply a microenvironment that is convenient for the exchange of nutrients *via* a porous structure and cell proliferation and have mechanical characteristics that approximately match natural tissue. The effect of the NCD content on the morphology structure, mechanical strength, swelling ratio, and biodegradation has been investigated. The results indicate that nanocomposite hydrogels containing a higher content of NCDs have smaller pore sizes and higher mechanical properties. The *in vitro* biodegradation and swelling behavior demonstrated that increasing the amount of NCDs up to 0.06% decreased the swelling ratio and weight loss of the hydrogels. The composite hydrogels are biocompatible, as verified by the MTT assay of MG-63 cells. The N-doped graphene quantum dots considerably affect degradation and interaction within the cells and hydrogels.

## Abstract 2

The low gelation time (120 s) and gelation temperature at body temperature (37 °C) make oxidized alginate/gelatin/NCDs hydrogels suitable as temperature-sensitive injectable hydrogels for cartilage tissue engineering.

## Graphical abstract

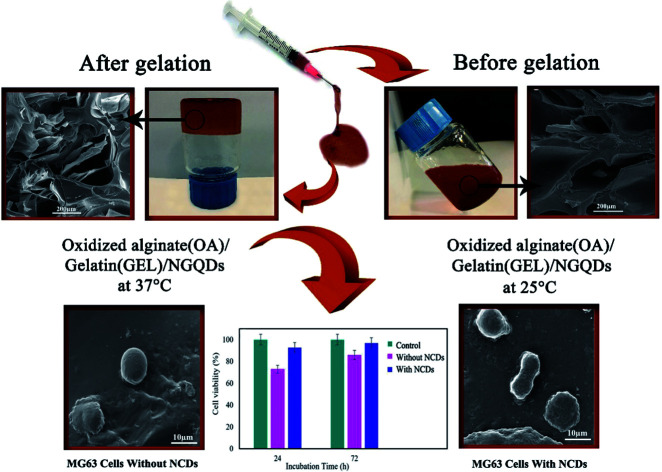

## 1. Introduction

Tissue engineering, which is a combination of life sciences and engineering procedures, provides a novel method to fabricate an architectural and practical biological replacement for fixing issues with the role of tissue within the usage of bioactive molecules, cells, and scaffolds.^1–3^ Hydrogels are one of the fundamental classes of biological materials used for the fabrication of scaffolds due to their comparable compositions and mechanical behavior to the extracellular matrix (ECM) of native cartilage tissues, high interaction with cells, and sufficient preparation of signals to maintain cell attachment, generation, and distinction.^4,5^ Hydrogels are 3D networks of cross-linked hydrophilic polymer chains, which absorb an amount of water more than a thousand times their dry mass, creating a jelly form that mimics the stem-cell niche. They are classified as synthetic and natural polymer-based hydrogels depending on their sources of raw material. Among them, natural hydrogels are designated as a promising subject of current investigation owing to their excellent biological characteristics.^4,6^ Injectable hydrogels are the latest research in this field. Besides this, they are non-toxic and easy to prepare. Hydrogel stability is a notable benefit that can be prepared *via* different physical and chemical cross-linking procedures.^7,8^ Lately, the interplay of the *in situ* chemical cross-linking within macromolecules utilizing the Schiff base reaction between amine groups and aldehyde groups has been investigated because of multiple advantages, including reversibility, simplicity, easy control of reaction rate below moderate states, pH-sensitivity, biocompatibility, and avoidance of chemical crosslinkers.^6,8^ Hydrogels possess a great potential for cartilage repair because of their adjustable mechanical and chemical features. Therefore, a hydrogel scaffold can adjust cellular activity and bring about hyaline cartilage tissue regeneration.^9,10^

The sodium salt of alginic acid or alginate is a natural polysaccharide obtained from brown algae. Alginate is composed of alpha-l-guluronic acid and beta-d-mannuronic acid units, which are organized in poly(alpha-l-guluronate) (G blocks) and poly(beta-d-mannuronate) (M blocks).^11^ Since alginate has no adhesive ligands to activate cell attachment, a biopolymer, preferably a protein, must be combined to activate cell-material interplay.^12^ Gelatin is a biodegradable natural polymer, resulting from hydrolysis of collagen and cleavage of the triple helix. As collagen is a major component of the extracellular matrix (ECM), collagen, and gelatin are broadly applied in medical applications and tissue engineering.^11,12^ Gelatin can be combined with alginate *via* forming covalent bonds,^13^ by which alginate must be oxidized in a monitored way within potassium periodate (KIO_4_) to create oxidized alginate (OA). The amino group residuum hydroxylysine or lysine of gelatin are covalently linked by the formed aldehydes of OA within the Schiff base reaction in the OA–GEL hydrogel.^12^ Since gelatin liquefies at normothermia temperatures, it is essential to crosslink it to generate a temperature-stable hydrogel. However, these hydrogels have low rheological properties, which limit their utilization. The addition of nanostructures in OA–GEL is an innovative advancement. It is hypothesized that uniform nanostructures can enhance intermolecular hydrophobic interactions by combining nanocomposite hydrogels and improve the rheological behavior of the hydrogels. Also, nanostructures can augment the OA–GEL network structure, providing enhanced thermal and mechanical characteristics. So far, couples of inorganic components have been considered, which incorporated metal oxide NPs,^14^ layered double hydroxides,^15–17^ carbon nanotubes,^18^ hydroxyapatite,^19,20^ graphene oxide,^21,22^ and clay minerals.^23–25^

We have chosen materials that can simulate cartilage properties: oxidized alginate (OA), gelatin (GEL), and nitrogen-doped carbon dots (NCDs) as reinforcement. OA is a biodegradable hydrogel that can mimic the extracellular matrix (ECM). GEL is also biocompatible in medical procedures. NGO possesses several benefits, for instance, great biocompatibility, excellent environmental durability, inexpensive fabrication, and earth-abundant raw materials.^26^

In this paper, hydrogel composites including, OA/GEL/NCDs were prepared by crosslinking the amino groups of GEL and the aldehyde groups of OA using *N*-hydroxysuccinimide (NHS) and 1-ethyl-3-(3-dimethylaminopropyl) carbodiimide (EDC) as chemical crosslinkers. We investigated the influence of the oxidation of alginate on the mechanical, physical, and morphological properties and the cytotoxicity of this hydrogel. We anticipate that this hydrogel creates a biodesign microenvironment with high biodegradation and biocompatibility for repairing cartilage tissue.

## 2. Materials and methods

### 2.1. Materials

Gelatin (microbiology grade), sodium alginate (molar mass 10 000–600 000 g mol^−1^), *n*-propanol, potassium periodate, ethyl alcohol, diphenyl ether, sodium chloride, acetone, ethylene glycol, 1-ethyl-3-(3-dimethylaminopropyl) carbodiimide (EDC), hexane, *N*-hydroxysuccinimide (NHS), *p*-phenylenediamine and silver nitrate were purchased from Merck company and utilized without further purification.

### 2.2. Oxidation of alginate

2.01 g of sodium alginate and 11.2 mL of *n*-propanol were blended with DI-water in a 250 mL beaker to obtain 225 mL in total. The mixture was kept at 30 °C in the dark under stirring (5 h) to dissolve the alginate completely. 1.16 g of potassium periodate (KIO_4_) dispersed in 30 mL DI-water was combined with the alginate solution. The mixture was kept in the dark for 24 h. The reaction was quenched by adding 1 mL of ethylene glycol (EG) and the mixture was agitated for another 30 min. 6.5 g of sodium chloride (NaCl) was dissolved in the above suspension to purify the polymer, which was next gradually added to 400 mL agitated ethyl alcohol. The white precipitate was dissolved in DI-water with 3.3 g of NaCl and reprecipitated in 250 mL ethyl alcohol. The precipitate was dissolved in DI-water again and precipitated in 200 mL acetone. Eventually, the precipitate was rinsed in agitated ethyl alcohol for 15 min, refined, and dried at 25 °C.^27^ The lack of periodate was controlled by combining 500 μL fractions of the dialyzate to 500 μL of a 1% silver nitrate solution, assuring the nonexistence of any precipitate.^28^

### 2.3. Fabrication of N-doped carbon dots (NCDs)

N-doped carbon dots were synthesized through the method reported by Wang *et al.*^26^ In a typical procedure, 0.2 g of *p*-phenylenediamine (PPD) dissolved in 15 mL of diphenyl ether was heated at 200 °C for 3 h and next chilled to ambient temperature. The mixture was added to 50 mL of hexane to the precipitate. The total mixture was then centrifuged at 5000 rpm for 10 minutes. After redoing the precipitation and centrifugation procedure three times, a brown powder was collected.

### 2.4. Preparation of the OA/GEL/NCDs hydrogels

5 mL of 6 wt% of OA solution was agitated with 5 mL of 15 wt% of GEL at 37 °C. The cross-linker, including a mixture of 0.1 g EDC and 0.05 g NHS, was added to the above solution. The first gelation was observed in 4–5 s and kept at 37 °C, resulting in the creation of a perfect gel after 2 min.

Different weight percentages of NCDs (0.06%, 0.04%, and 0.02%) were added to the 5 mL of 6 wt% of OA solution and agitated for 5 min. Next, 5 mL of 15 wt% of GEL was added to the suspension and stirred for another 5 min. The final solutions were mixed for 2 min by adding EDC and NHS as cross-linker agents. The samples were freeze-dried for 24 h.

### 2.5. Swelling ratio and biodegradation

The water absorption of the hydrogels was evaluated by the gravimetric technique. About 0.3 g (*W*_0_) of the hydrogels was incubated in 10 mL PBS for 24 h to attain equilibrium swelling. The buoyant was removed and the swollen hydrogels were weighed (*W*_s_). The swelling ratio (SR) was expressed by the following equation:^19^1
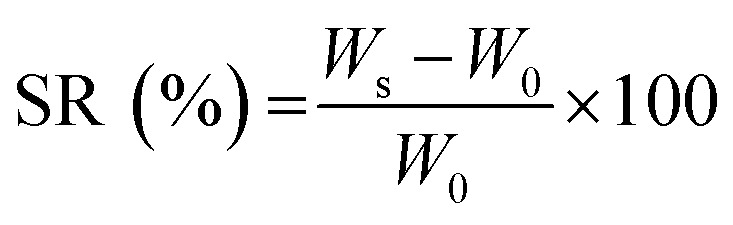
mass degradation/erosion degrees were additionally evaluated likewise at various periods up to 21 days. All tests were accomplished three times.

### 2.6. Materials characterization

Fourier transform infrared spectroscopy (Shimadzu Varian 4300 spectrophotometer) was utilized to investigate the chemical composition of oxidized alginate and the fabricated hydrogels by applying KBr pellets in the wavenumbers between 4000–400 cm^−1^. Field emission scanning electron microscopy (TESCAN MIRA 3 FE-SEM) was used to study the morphological and structural properties of the lyophilized hydrogels. The lyophilized hydrogels were cross-sectioned, coated with gold (Au), and detected by FE-SEM at an accelerating voltage of 15 kV. High-resolution transmission electron microscopy (EM 208, Philips HR-TEM with an accelerating voltage of 100 kV) was utilized to observe nitrogen-doped graphene quantum dots. A Physica MCR 300 Rheometer (Anton Paar Ltd., Austria) was utilized to measure the oscillatory rheological properties of the hydrogels.

### 2.7. Mechanical properties

A Physica MCR 300 Rheometer (Anton Paar Ltd., Austria) was used to measure the rheological attributes of the hydrogels utilizing a circular disk parallel plate with a diameter of 25 mm and a gap of 0.5 mm. An amplitude sweep was conducted at a consistent angular frequency of 1 Hz to define the limit of linear viscoelasticity. The strain amplitude was kept at 0.1% during the test. The contributions of the liquid-like form (viscous modulus (*G*′′)) and solid-like form (elastic modulus (*G*′)) were noted through temperature sweeps from 20 to 50 °C at a speed of 1 °C min^−1^ to assess the thermogelling attributes (angular frequency = 1 Hz). Each following rheological test was conducted below the simulated physiological states (in PBS pH = 7.4 at 37 °C), considering the possible utilization of the hydrogels. The oscillatory rheological determination as a function of time was conducted at a consistent frequency of 1 Hz to evaluate the time of gelation. The gel point or gelation time was specified as the time that the loss modulus and shear storage modulus were identical.^29^ The hydrogels were swollen for 1 h in 1 mL PBS and moved to the rheometer stage for performing crosslinked hydrogels. Next, frequency sweep analyses in the linear viscoelastic area were performed to determine the dynamic viscoelasticity at 37 °C at a broad range of frequencies (0.1–100 Hz).

### 2.8. *In vitro* biological assays

The *in vitro* biocompatibility of the hydrogels was estimated by utilizing 3-(4,5-dimethylthiazol-2-yl)-2,5-diphenyltetrazolium bromide (MTT assay), which depends on the mitochondrial MTT reduction to produce an insoluble dark blue formazan. The samples were incubated in 1 mL of RPMI 1640 culture medium (Sigma-Aldrich) at 37 °C supplied by 10% (w/w) fetal bovine serum (FBS) for 24 and 72 h to achieve the extracts of the as-dried hydrogels. The growth medium (RPMI and FBS) was utilized as the control under similar conditions. The MG63 cells were cultivated in 96-well plates at a density of 1 × 10^4^ MG63 cells per sample. The growth medium was substituted by the hydrogels extract. The extract was removed after 24 h. 100 μL of the MTT solution (0.5 mg mL^−1^) was added to all wells and incubated for another 4 h at 37 °C. Then, the solution was eliminated and 100 μL isopropanol was consequently added to liquefy the MTT crystals. The absorbance of the solutions was measured with a microplate spectrophotometer (Biotek Powerwave XS2, USA) at 570 nm.

In order to study the architecture of the cell attached to the hydrogels, cross-sectional SEM images of the samples were recorded. The hydrogels were put in a Petri dish and incubated in the presence of DMEM and MG63 cells at 37 °C for 24 h. After incubating, the hydrogels were rinsed multiple times with PBS and set by 2.5% glutaraldehyde solution for 4 h at 4 °C. Eventually, the samples were lyophilized and coated with Au for the FESEM surveys.

## 3. Result and discussion

**Fig. 1:**
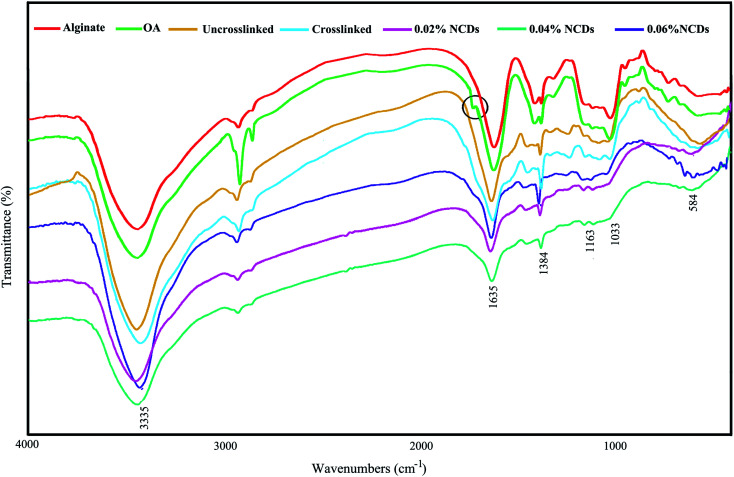
FTIR spectra of alginate, oxidized alginate, and the hydrogels.

A new faint peak was recognized at 1732 cm^−1^ in the spectrum of OA (Fig. 1), which was assigned to the symmetric vibration of the aldehyde group, showing that alginate is effectively oxidized *via* the KIO_4_. Besides, a decreased severity in the absorption was perceived at 815 cm^−1^ in the OA. It might have led to the C–O–C decomposition on the alginate chains after the oxidation.^30^ The spectra of the hydrogels show characteristic peak bands at 1635 cm^−1^ for the C=N stretching vibrational mode, implying Schiff base reactions. The peak at 1635 cm^−1^ is wide due to overlaying with an amide-I group of the uncrosslinked GEL at 1632 cm^−1^.^30^ The characteristic bands at 1035 and 1163 cm^−1^ are designated as C–O, and C–O–C vibrational modes in the mannuronic and guluronic groups, respectively. The bands at 1635 and 1384 cm^−1^ are correlated to the carboxylic acid units of alginate. The broad peak at 3335 cm^−1^ is related to –NH and –OH stretching modes. The bonds of the NCDs overlap with the peaks of alginate and gelatin and are not detectable in the spectrum due to the low amounts of NCDs in the hydrogels.

**Fig. 2:**
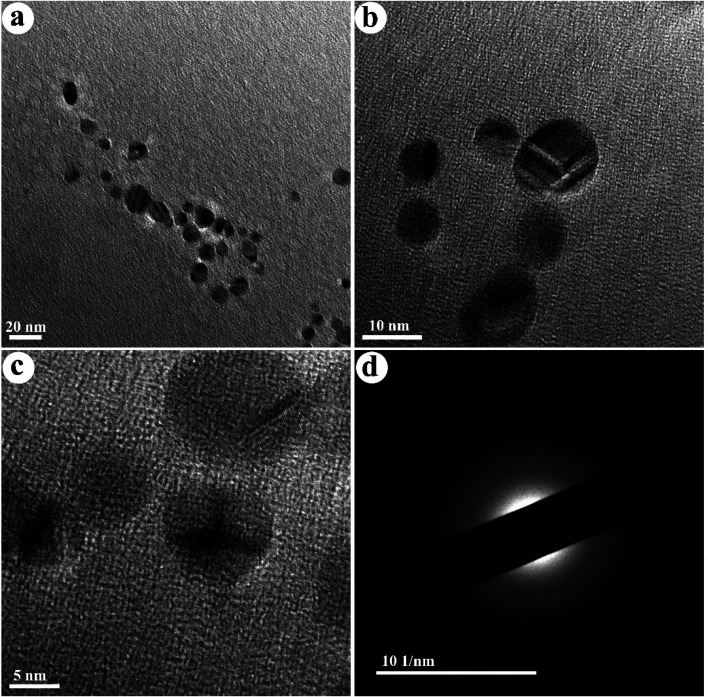
(a–c) TEM images of NCDs and (d) SAED of NCDs.

The TEM images of the as-fabricated N-doped carbon dots (NCDs) are shown in Fig. 2. The NCDs with a particle size ranging from 6 to 14 nm and an average diameter of ≈9 nm are shown in this figure. Also, their distribution is monodisperse and regular. The SAED pattern presented in Fig. 2d shows diffused rings, indicating the polycrystalline nature of the synthesized NCDs.

**Fig. 3:**
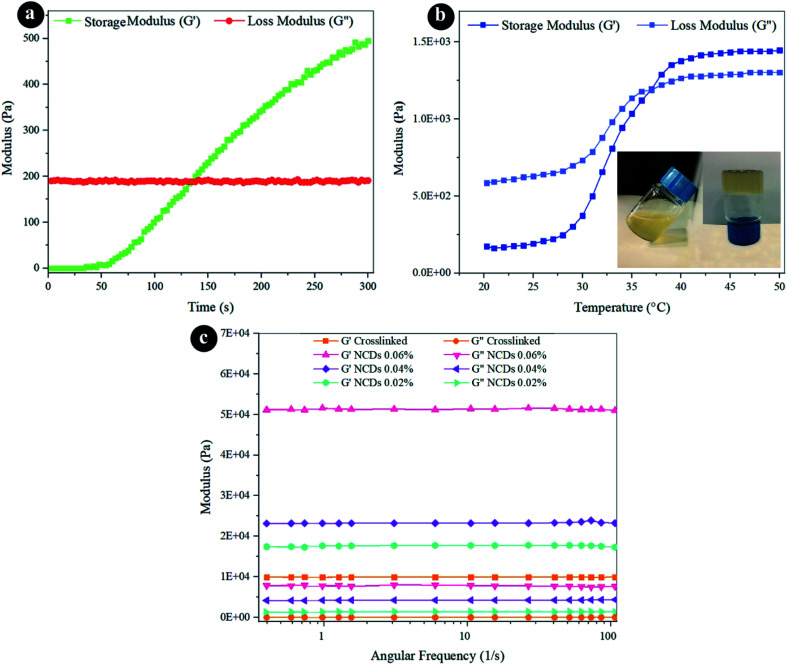
Rheological properties of the hydrogels by (a) time sweep, (b) temperature sweep, and (c) frequency sweep.

The gelation time is one of the most crucial factors of injectable hydrogels for their utilization. The long gelation time results in an irregular distribution of substances. The rheometer was selected to control the changes of loss modulus (*G*′′) and storage modulus (*G*′) with the time progression in the liquid to solid transition phase after adding crosslinkers. The preceding *G*′ was less than *G*′′, but the growth rate of *G*′ was notably greater than *G*′′. Wherever the *G*′ crossed the *G*′′, this time location was described as gelation time. As indicated in Fig. 3a, before gelation time, the *G*′ was less than the *G*′′, showing the liquid phase of the substance. After the gelation time, the *G*′ was higher than the *G*′′, which indicates that the hydrogel possesses a solid phase. The gelation time of the hydrogel is 120 s, which is adequate for performing as an injectable hydrogel.

The thermosensitive behavior of the crosslinked hydrogel was distinguished by utilizing a temperature sweep analysis, as presented in Fig. 3b. *G*′′ was higher than *G*′ at lower temperatures, implying a fluid-like behavior. *G*′ improved notably quicker than *G*′′ during the heating process. At higher temperatures, *G*′ became much bigger than *G*′′, designating a jelly behavior. The intersection within *G*′ and *G*′′ at 37 °C was recognized as the gelation temperature.^31^ This implies that human body temperature can procure the alteration of the fluid to a gel.

The mechanical characteristics of the hydrogels perform the main role in their utilization, which concludes whether they can remain in a specific region and have an impact. *G*′ indicates the strength of hydrogels to store elastic deformation energy and indicates the hardness of the hydrogels. A rheometer was applied to identify the alterations of *G*′ and *G*′′ *vs.* angular frequency. As presented in Fig. 3c, the *G*′ was greater than the *G*′′ in the whole frequency spectrum, which indicates that the elasticity is dominant. It expressed that the frequency changes did not damage the composition of the hydrogels and that the hydrogels retained a solid phase. Also, the *G*′ stayed constant amongst the entire frequency spectrum, confirming the character of the crosslinked hydrogels. *G*′ developed quickly by increasing the quantity of NCDs, as illustrated in Table 1 and Fig. 3c. The *G*′ of the hydrogel with 0.06% NCDs was five-fold greater than the *G*′ of the crosslinked hydrogel. Expanding the amount of NCDs can lead to a greater crosslinking structure since it develops mechanical performance and the gelation through the existence of various reactive parts.

**Table 1:** Rheological properties of the hydrogels at 37 °C and a frequency of 1 Hz

Sample	Storage modulus (Pa)	Loss modulus (Pa)	Average pore size (μm)
Crosslinked	9929 ± 24	11.5 ± 0.3	161.1
0.02% NCDs	17 515 ± 365	1278 ± 106	116.9
0.04% NCDs	23 425 ± 465	4247 ± 124	115.3
0.06% NCDs	51 185 ± 376	7730 ± 233	102.5

**Fig. 4:**
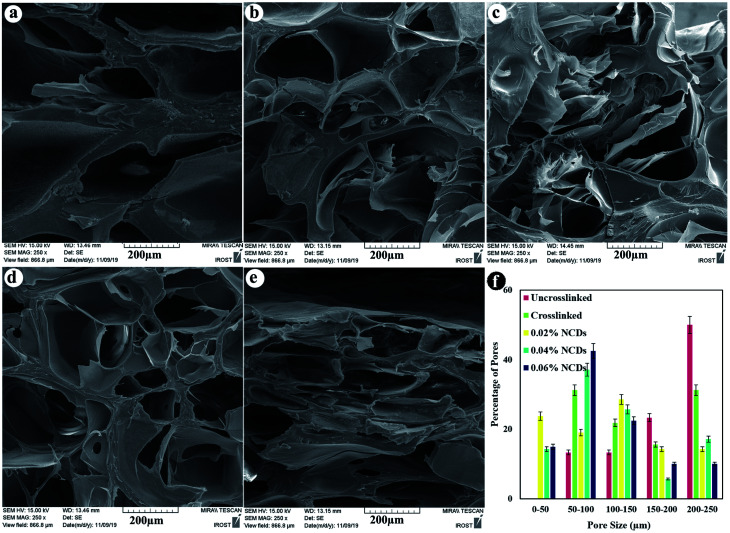
Cross-sectional morphology of freeze-dried hydrogels (a) uncrosslinked, (b) crosslinked, (c) and containing 0.02% NCDs, (d) 0.04% NCDs, and (e) 0.06% NCDs. (f) The size distribution diagram of the samples.

Cross-sectional FESEM images were taken to identify the lyophilized hydrogel microstructures (Fig. 4). The hydrogels exhibited a porous architecture due to the freeze-drying step, which produced pores as a result of the formation of ice crystals and is similar to other natural macromolecular hydrogels, with average pore sizes ranging from 102 to 161 μm. The interior morphology depended on the ratio of the NCDs contents. A greater ratio of NCDs leads to smaller pore diameters.

**Fig. 5:**
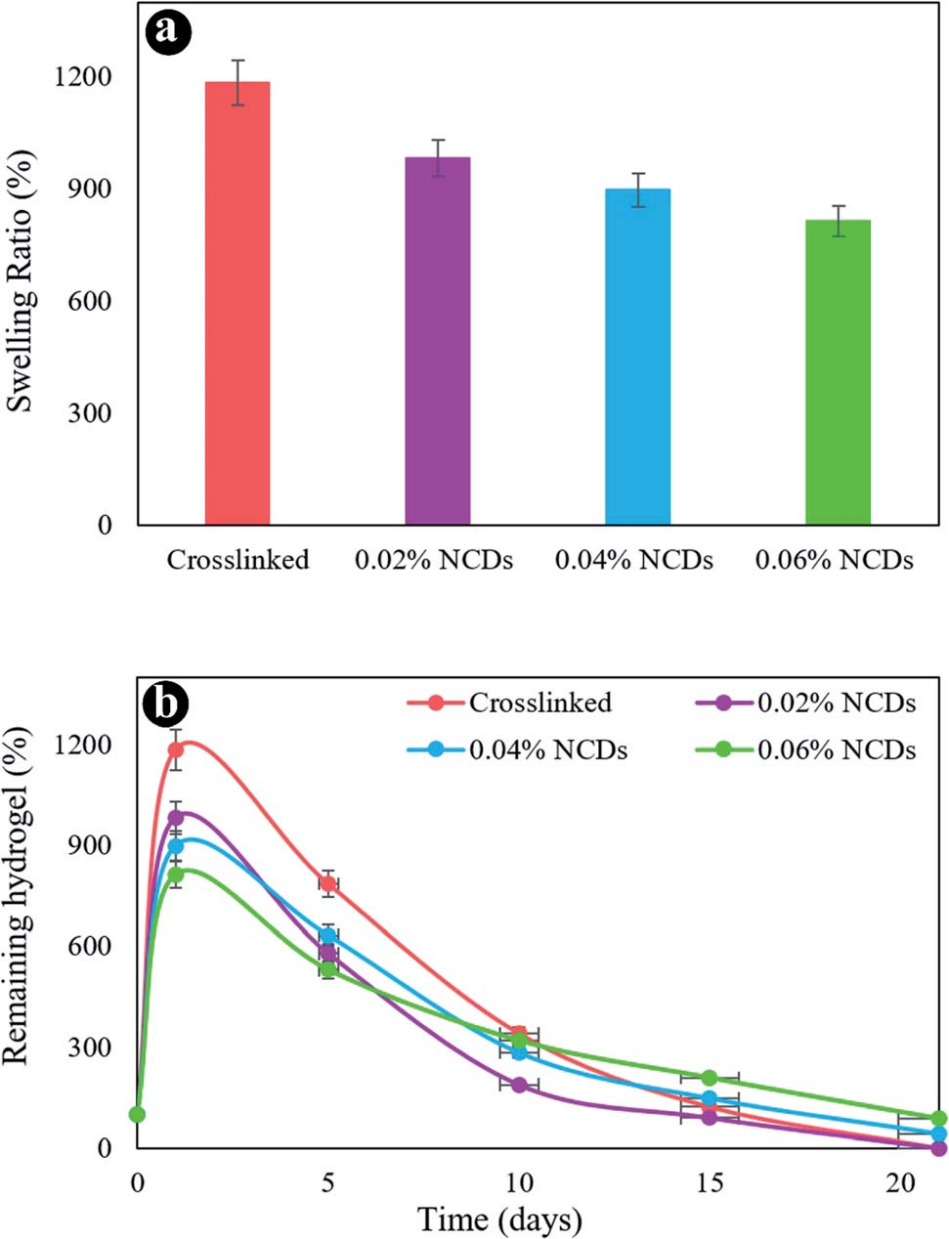
(a) The swelling ratio of the hydrogels and (b) *in vitro* biodegradation after various incubation times in PBS at 37 °C.

The swelling behavior of the samples was assessed in PBS at 37 °C. Fig. 5a revealed the swelling ratio of the hydrogels after 24 h. The swelling capacity of the hydrogels diminished with an increment in NCDs content. The crosslinked hydrogel exhibits a swelling percentage of 1185% in 24 h. The hydrogels containing 0.02% to 0.06% of NCDs revealed a swelling degree reducing from 983 to 813%, respectively. A small reduction was observed in the swelling rate by enhancing the amount of NCDs because of the strong interplay between the NCDs and the hydrogel system.

Fig. 5b reveals the mass loss of hydrogels as a function of incubation time. The first weight increment was assigned to the swelling behavior of the hydrogel system, providing higher water absorption. The increase in the NCDs content notably reduced the degradation ratio of the hydrogel after 21 days. On day 21, the residual percentage of 0.06% NCDs and 0.04% NCD hydrogels was 86% and 43%, respectively. By increasing the quantity of NCDs in the hydrogel network, hydrogel structures were demonstrated to be more stable than others. The presence of N-doped graphene quantum dots led to an increased crosslinking denseness, which affected several obvious features of the hydrogels, including enhanced mechanical properties, dense microstructures, slower weight loss, and less water uptake. We chose the nanocomposite hydrogel containing 0.06% NCDs as a perfect sample for additional research for consideration as a cartilage scaffold.

**Fig. 6:**
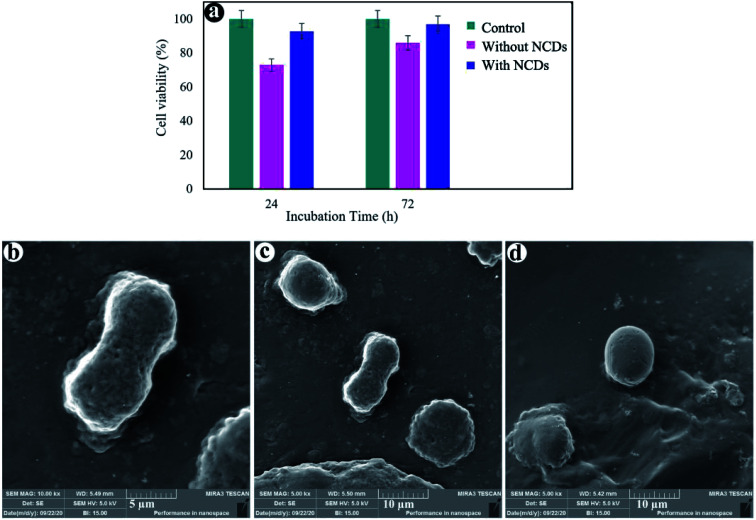
Cell viability (a), FE-SEM images of the cell-cultured hydrogels with 0.06% NCDs (b and c) and without NCDs (d).

A perfect biomedical substance should not produce adverse reactions or release toxic products, and it could be assessed within *in vitro* cytotoxicity assays. The MTT assay is commonly acknowledged as a standard technique for determining the toxicity of materials. The effect of hydrogels with and without NCDs on the viability of MG63 cells was examined *via* an MTT assay after 24 and 72 h of incubation (Fig. 6a). The cell viability of the hydrogel without NCDs was 73% and 86% after 24 and 72 h, respectively. Remarkably, the cell viability of the hydrogel containing 0.06% NCDs was improved after 1 and 3 day incubation (92.8% and 97%) due to the presence of NCDs in the hydrogel. The hydrogel with NCDs can be interiorized by the cells; hence, the MG63 cells can attach more effectively to the hydrogel matrices in the presence of the NCDs. The outcomes recommend that the hydrogel with 0.06% NCDs presents a guarantee as an injectable hydrogel that can be applied in cartilage regeneration or tissue engineering. Fig. 6b–d indicates the morphology of the MG63 cells on the surface of the hydrogel with and without NCDs after 24 h by scanning electron microscopy. Cell attachment and distribution on the scaffolds were investigated by SEM. The hydrogel containing 0.06% NCDs reveals higher cell adhesion. The cells attached to hydrogel with NCDs were observed to be greater in number, well distributed, and well-attached to the surface compared to the ones without NCDs. Cells seeded on the hydrogel without NCDs were spherical without any remarkable interplay within the hydrogel and cells. However, the NCDs hydrogel promoted cell adhesion, enabling the cells to be completely distributed on its surface and to attach to other cells.

Table 2 reviews the different hydrogels and their properties obtained for tissue engineering. As demonstrated in this table, OA/GEL/NCDs composite hydrogels are comparable with other composites reported in the literature.

**Table 2:** Different scaffolds and their properties obtained for tissue engineering

Materials	Cells	Cell culture	Special	Ref.
Alginate–PVA–hydroxyapatite	Mouse calvaria (MC) 3T3-E1	14 days	*In vitro*, viability = ∼77% (after incubation), >∼22% for only alginate, could maintain structure for at least 14 days. Alginate hydroxyapatite had optimum mechanical, rheological and biological characteristics. PVA–hydroxyapatite modulates viscosity and thus enhanced osteoconduction and viscosity	32
Alginate–polylactic acid short fibers	Human chondrocytes	14 days	*In vitro*, ∼80% viability	33
Alginate–PVA–hydroxyapatite–collagen	Mouse calvaria (MC) 3T3-E1	10 days	*In vitro*, initial viability >98%, collagen increases cell adhesion and activity	34
Na alginate–collagen or Na alginate–agarose	Chondrocytes from the articular cartilage of rats	14–21 days	Na alginate–collagen possessed better mechanical strength and bioactivity than other combinations	35
Alginate–polycaprolactone	Human nasal septum cartilage chondrocytes	7 days	Osteogenic tissue engineering, viability = ∼94% (chondrocytes) ∼96% (osteoblast), no observable proliferation in chondrocytes	36
Alginate–alginate sulfate	Bone morphogenetic protein-2, MC3T3-E1	7 days	*In vitro*, 80% proliferation, improved retention of proteins, alginate–alginate sulfate tightly bound BMP-2, which aids adhesion and cell viability, Ca presence and porosity favorable for bone tissue engineering	37
Alginate–gelatin–fibrinogen	Glioma cells/stem cells	21 days	3rd week showed accelerated growth mimicking the tumor spreading and growth, cell viability = 86.92%	38
Sodium alginate–gelatin	Rat Schwann cell line (RSC96)	14 days	Viability = ∼93% (post 14 days), printed structures start degrading after 14 days	39
Alginate–polycaprolactone	Chondrogenic cell ATDC5	21 days	*In vivo*, ∼70 viable cells, maintained integrity of the PCL scaffold even after 21 days, composite mimics the natural characteristics of cartilage	40
Oxidized alginate–gelatin–NCDs	Osteosarcoma cell line MG63	1–3 days	Viability = 97% after 3 days, adding NCDs increases cell viability, cell attachment, and storage modulus	This work

## 4. Conclusions

Currently, several new substances and medical procedures are helping to repair damaged cartilage tissue. In summary, biodegradable and injectable OA/GEL hydrogels comprising different quantities of NCDs have been successfully fabricated and crosslinked by EDC/NHS as chemical crosslinkers. Variation in the amount of NCDs affected the mechanical properties, water uptake, biodegradation, and pore sizes. The results revealed that the hydrogel containing 0.06% NCDs possessed smaller pore sizes (102 μm), lower water uptake (813%), better mechanical properties, and less weight loss due to the strong interplay between NCDs and the hydrogel system. The temperature and time sweep showed that human body temperature could change fluids to gel and the nanocomposite hydrogels are suitable for injection in cartilage tissue regeneration. The *in vitro* biodegradation demonstrated that the crosslinked hydrogel (without NCDs) was more unstable than the one with a higher NCD content. The nanocomposite hydrogel showed excellent cell adhesion to MG63 cells and a cell viability of >97% in the MTT assay. In general, the oxidized alginate/gelatin/NCDs nanocomposite hydrogels revealed excellent mechanical and stability properties by mimicking ECM biochemical markers, which could notably modify their biological performance in cartilage tissue engineering as injectable hydrogels.

## Conflicts of interest

The authors declare that there are no conflicts of interest regarding the publication of this manuscript.

## Supplementary Material
